# Knowledge, Attitude, and Practice Regarding Zika Among Travelers to Brazil: Qatar’s Airport Study 2017

**DOI:** 10.7759/cureus.3280

**Published:** 2018-09-10

**Authors:** Ayman Al-Dahshan, Aya Moustafa, Minahil Khalid, Mohamed Nour, John Roberts, Sheila Cantos, Hamad E Al-Romaihi, Salih Al-Marri, Mohamad A Chehab, Mueen Sharaf, Mohammed Al-Thani, Ahmed El-Sayed, Mohd Alhajri, Elmoubasher A Farag

**Affiliations:** 1 Preventive Medicine, Hamad Medical Corporation, Doha, QAT; 2 Epidemiology and Public Health, Ministry of Public Health, Doha, QAT; 3 Preventive Medicine, Ministry of Public Health, Doha, QAT; 4 Miscellaneous, Qatar Airways, Doha, QAT; 5 Miscellaneous, Hamad International Airport, Doha, QAT; 6 Family Medicine, Ministry of Public Health, Doha, QAT; 7 Community Medicine, Hamad Medical Corporation, Doha, QAT

**Keywords:** emerging, zika virus, travel-related illness, knowledge, attitude, qatar

## Abstract

Background

The Zika virus has become an international health issue and poses a systematic risk for a growing number of travelers. Qatar is no exception to this status, where its Hamad International Airport (HIA) has become an important hub for many travelers to and from affected countries. Thus, it is a national necessity to determine the knowledge, attitude, and practice of travelers’ regarding this emerging disease in the State of Qatar.

Methods

This was a cross-sectional study that employed a self-administered questionnaire (n=100) and was conducted at Hamad International Airport. Descriptive and inferential statistical tests were applied to analyze the data using the Statistical Package for the Social Sciences (SPSS) (IBM SPSS Statistics 21, IBM Corporation, Armonk, NY, USA, 2014).

Results

The majority of travelers (75%) reported hearing about the Zika disease prior to their current flight, mainly from the media (73%) and the internet (46%). The majority of participants (69%) knew about the vector-borne transmission and symptomatology of a Zika infection while more than half (54%) correctly identified effective methods to prevent infection. Regarding their attitude, less than two-thirds (58%) of the participants agreed that pregnant women must postpone their travel to any Zika-affected area. Regarding their practice, only a quarter of the sampled travelers (24%) sought pre-travel medical advice before going to Brazil. Comparing the knowledge score among different education levels, we found that high-school students scored significantly better than those with diplomas and bachelors, p=0.042 and p=0.012, respectively.

Conclusion

The survey findings revealed that the knowledge of Zika infection is low among travelers to Brazil. Thus, stronger efforts to educate travelers about Zika are recommended. It is also vital that travelers be encouraged to seek proper medical advice prior to travel.

## Introduction

Zika is a viral disease and is transmitted by the Aedes aegypti mosquito, preferably in warm urban environments [1]. The first reported cases of Zika among humans arose in Nigeria in 1954 [2]. The first major outbreak of Zika viral infection was recognized in Yap, Micronesia, in 2007, where almost 18% of infected individuals became symptomatic [3]. The virus is currently responsible for a globally emerging infectious disease that affects 84 countries and territories in the Caribbean and South and Central America [4]. Moreover, most of the epidemic and endemic viral transmission of Zika has been documented in tropical as well as subtropical regions [5]. The emergence of the Zika infection has been significantly associated with the appearance of certain neurological outcomes, such as neonatal microcephaly in Brazil and Guillain-Barré syndrome among adults in Polynesia [6-7].

According to international recommendations, it is advised that pregnant women or those planning to become pregnant avoid or even postpone their travel to areas with high transmission rates. If it is not possible, they should protect themselves from being bitten by mosquitoes through adequate precautions, such as bed nets, insecticide use, and window and door screens. In addition to that, men and women should embrace safe sexual practices, especially when residing in Zika-endemic areas [8]. Up to date, the World Health Organization (WHO) has not issued any recommendation against travel to the affected countries. Currently, there is no approved vaccine for Zika prophylaxis, but multiple vaccine trials are currently in process to develop an effective vaccine [9]. Treatment is symptomatic, as there is no specific medication for a Zika infection; however, travelers to regions of active transmission must pursue medical care once they experience any symptoms similar to the Zika infection.

Research has estimated that 2.6-billion people live in countries at risk of the introduction of a local Zika transmission. Furthermore, such risk is attributed to a variety of factors, including the presence of a competent mosquito vector, suitable environmental conditions, and a heavy influx of travelers from Zika endemic areas. Thus, travelers might serve as a driver behind the global spread of Zika, making them the ideal primary respondents against the disease [10].

In addition to that, community understanding of the risks associated with Zika transmission as well as the possible prevention measures is critical, especially among vulnerable groups such as women and children. A study conducted at university-based medical clinics revealed that only 10% of the men were concerned about the possibility of transmitting the virus to their sexual partners after returning from endemic areas. Other findings concluded that about half (55%) of the respondents restricted their travel to Zika endemic regions while the majority (95%) planned to adopt protective precautions against mosquitoes during their stay. Moreover, a small percentage (15%) of women planned to breastfeed while on travel to the endemic areas and a similar portion (16%) planned to refrain from sex if pregnant (Poster: Katler Q, Godiwala P, Macri C, Pineles B, Chang AI, Ahmadzia H: Healthcare Provider and Patient Knowledge Attitudes and Practices (KAP) Regarding Zika Virus. GW Annual Research Days; 2017).

A phone-based survey among women of childbearing age found that two-thirds of the participants reported taking some preventive actions while on travel to the affected areas, such as the use of mosquito repellents as well as wearing protective clothes and gloves. Also, the majority of the participants (84%) agreed that the infection can be asymptomatic and almost half (56%) knew about the possibility of transmission via sexual contact [11]. In fact, Zika has been reported to be transmitted via vertical transmission from mother to child, through breastfeeding, and sexually [12-14].

The Zika virus disease is a major international public health issue. Qatar is no exception to this status where its Hamad International Airport (HIA) has become an important hub for many travelers to and from affected countries [15]. In addition to that, Qatar Airways navigates flights to several countries affected by Zika. However, there is a paucity of evidence on the awareness of travelers from Qatar regarding the Zika infection. The current study sought to evaluate the knowledge, attitude, and practice regarding preventive measures against Zika infection among travelers at HIA. This study will be the first phase of a process aimed at assessing travelers’ health and vector-borne disease risks in the State of Qatar.

## Materials and methods

Study population and design

This was a cross-sectional study conducted at HIA in Qatar during 2017. The target population included all travelers departing by air from HIA. For the purpose of this study, all those traveling directly to Brazil (Sao Paulo) and waiting in the boarding area at HIA (including transient passengers and travelers from Qatar) were recruited.

The outcomes measured were pre-travel health advice and consultations, such as the source of pre-travel advice (family physician, travel health specialist, or others), knowledge of common Zika infection-related signs and symptoms (fever, muscle pain, conjunctivitis, and others).

The calculated sample size using Epi-info software (Centers for Disease Control and Prevention (CDC), Atlanta, Georgia, US) resulted in 99 travelers, using a 95% confidence level, a 7% standard error, and assuming a 50% prevalence of pre-travel advice and consultations (taken as the most conservative value, as there is no previous study done among travelers in Qatar). Then, a non-probability convenient sampling technique was employed [16]. This sampling method was chosen because it was not feasible to obtain a list of all the travelers heading to Brazil at HIA.

The current study followed the ethical standards of the Helsinki Declaration. Ethical approval was taken from the relevant authorities at the Ministry of Public Health (MoPH) in Qatar as well as security clearance from HIA. Only travelers who willingly gave verbal consent were interviewed. All personal information was handled with strict confidentiality. The researcher and data collectors were permitted to conduct this study in the premises of HIA (as part of the MoPH research team).

Data collection methods

Data collectors were recruited and trained prior to the launch of this study. On several visits, they were stationed at the departure lounge of the flight; after the passengers had checked in. Participants were selected from the waiting lounge until the required sample size of 100 was achieved. Each participant received a leaflet with information on Zika prevention after completing the questionnaire.

The data were collected from consenting participants through a validated, structured, and self-administered questionnaire, which included four main sections. The initial section of the questionnaire elicited data about the participants’ socio-demographic characteristics, medical conditions, travel-related history, and information on the current itinerary. The remaining sections encompassed nine questions, which were in multiple-choice format and distributed as four, three, and two questions related to the knowledge, attitude, and practice (KAP) of travelers regarding Zika infection, respectively. The development of the questionnaire was based on a literature review as well as experts' opinion to include previous evidence regarding the epidemiology of Zika infection as well as of the knowledge, attitude, and practice of travelers regarding this matter.

So, an English-based questionnaire was developed and validated through face validation. Subsequently, the questionnaire was distributed to experts in the field, including community medicine consultants and public health specialists at MoPH, and any relevant modifications were made. Afterward, the questionnaire was piloted, issues identified in this exercise were addressed in the final version.

Statistical analysis

Data were entered and analyzed through the Statistical Package for the Social Sciences (SPSS) software (IBM SPSS Statistics 21, IBM Corporation, Armonk, NY, USA, 2014). Descriptive statistics were employed as appropriate, such as proportions and percentages for categorical variables or means and standard deviation for continuous variables. The T-test and chi-square analysis were used to test for the difference between groups. All p-values less than or equal to 0.05 were considered statistically significant. A scoring system was followed for determining the level of knowledge among participants.

## Results

Following a response rate of 85%, a total of 110 travelers consented to participate and received the questionnaires. Upon a revision of the filled questionnaires, 10 were omitted, as they were incomplete. The participants were equally distributed between the different age groups and the gender distribution showed a predominance of males (68%). Also, about two-thirds of the respondents reported attaining a bachelor’s degree as their education level and the vast majority (93.9%) conveyed no comorbidities. As anticipated, the majority of travelers were found to be of Brazilian nationality (37%), given the flight destination to São Paulo. Filipinos were the second most common nationality (23%), followed by the Indian (7%), Chinese (7%), and Lebanese (7%) nationalities (Table 1).

**Table 1:** Demographic characteristics of the surveyed travelers (n=100)

Characteristics		Percentage (%)
Age (Binned)	<= 26.00	34.4%
	27.00 - 39.00	33.3%
	40.00+	32.3%
Gender	Male	68.0%
	Female	32.0%
Pregnant	Yes	0.0%
	No	100.0%
Education Level	High School	20.4%
	Diploma	6.8%
	Bachelors	64.8%
	Postgraduate	8.0%
Marital Status	Married	41.0%
	Single	57.0%
	Divorced	2.0%
Diabetes	Yes	1.0%
	No	99.0%
High Blood Pressure	Yes	4.0%
	No	96.0%
Heart Disease	Yes	0.0%
	No	100.0%
Other Disease	Yes	1.0%
	No	99.0%
No Disease	Yes	93.9%
	No	6.1%
Nationality	Brazilian	37.0%
	Pilipino	23.0%
	Others	19.0%
	Chinese	7.0%
	Indian	7.0%
	Lebanese	7.0%

Knowledge

**Figure 1:**
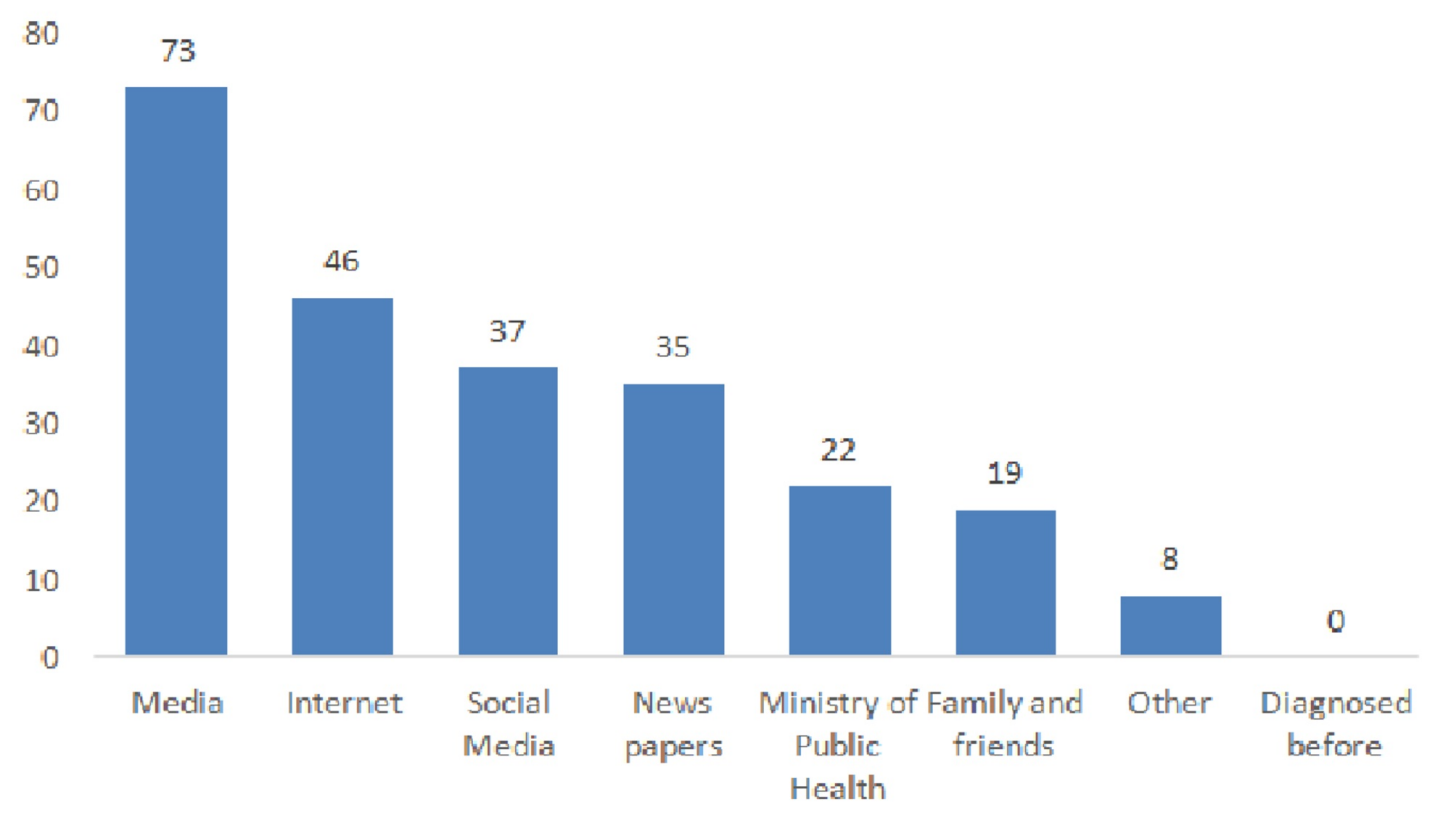
Distribution of travelers’ responses to the question: How did you hear of the Zika virus? (%)

Three-quarters of the participants (75%) had heard of the Zika viral disease prior to this travel. Among those who had heard about Zika, a large portion (73%) reported that the media was their source of information followed by the internet (46%) (Figure 1). Regarding the method of transmission, more than two-thirds (69%) correctly identified the mosquito bite as the main source of Zika viral transmission. On the other hand, many travelers were unaware of other sources of transmission as that from mother to child (19%) or through sexual contact (13%).

**Figure 2:**
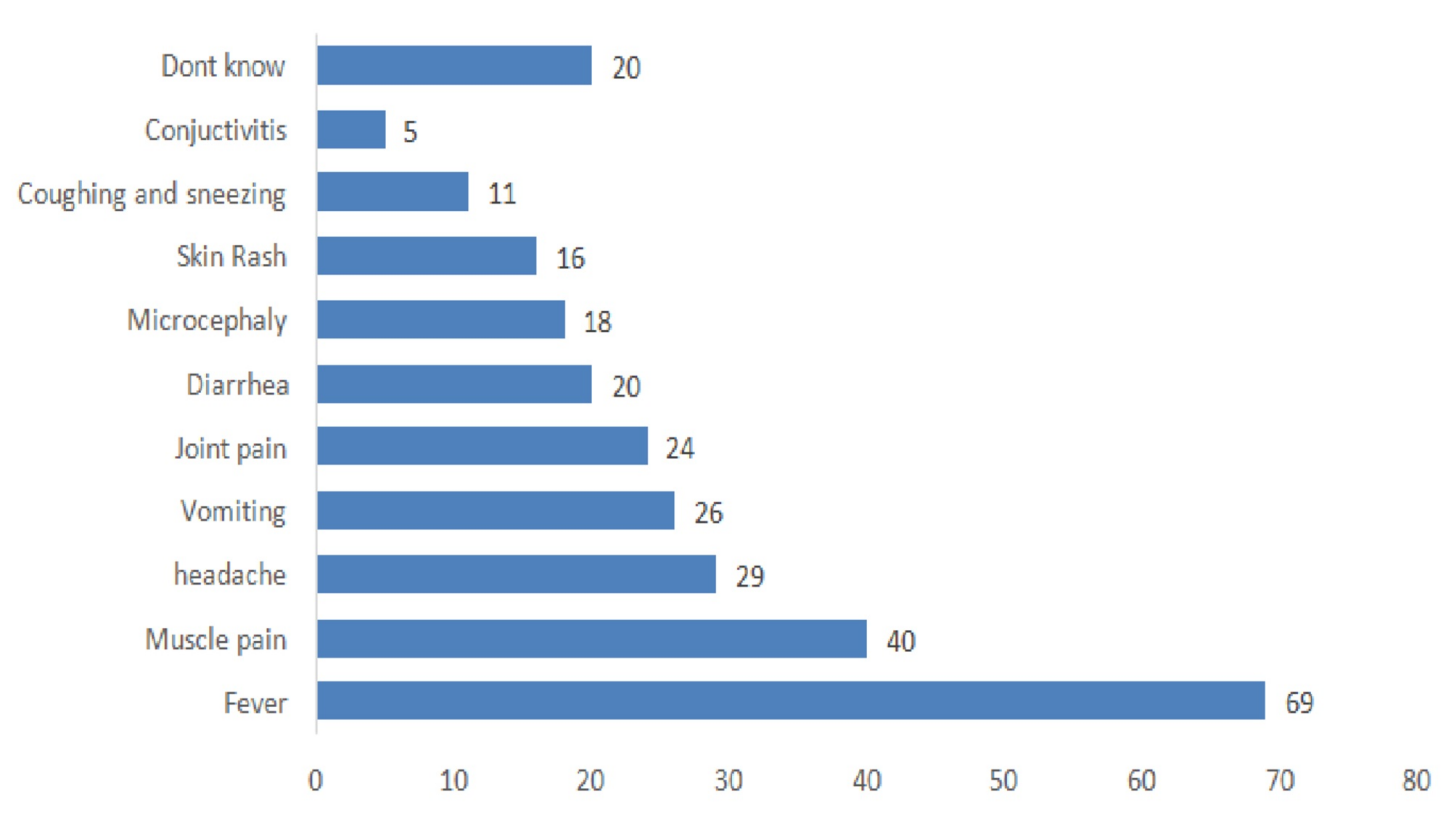
Traveler’s knowledge of symptoms/signs occurring during a Zika viral infection (%)

Regarding the participants’ knowledge of Zika infection’s symptomatology, more than two-thirds (69%) of respondents correctly identified fever as an associated symptom. However, many failed to identify other significant symptoms such as skin rash (16%) and conjunctivitis (5%). In addition, one-fifth (20%) of the surveyed travelers were completely unaware of the signs and symptoms associated with Zika viral infection (Figure 2).

More than half (54%) of the sampled travelers correctly identified effective preventive measures and reported that using mosquito repellents and eliminating standing water were successful methods to avoid a Zika infection. Nonetheless, some travelers had chosen ineffective prevention techniques, such as using antibiotics (11%), taking travel-related medication (17%), and wearing a mask (24%).

Upon a further analysis of the demographic characteristics and mean knowledge scores, it was found that those pursuing postgraduate studies and high-school students scored significantly better on the knowledge component of the questionnaire than those with a diploma or bachelor’s degree (p=0.024). Another finding was that travelers of Brazilian nationality were the most knowledgeable about the Zika infection and scored the highest mean knowledge score at 6.16 (p=0.005) (Table 2).

**Table 2:** Comparison of demographic characteristics and mean knowledge scores among participants (n=100)

Characteristics		Knowledge Score		p-value
		Mean	Standard Deviation	
Age (Binned)	<= 26.00	5.88	2.89	
	27.00 - 39.00	4.84	3.21	0.297
	40.00+	6.13	4.11	
Gender	Male	5.18	3.69	0.418
	Female	5.78	2.94	
Pregnant	Yes	.	.	
	No	5.37	3.46	
Edu. Level	High School	7.28	2.70	0.024
	Diploma	4.00	3.29	
	Bachelors	4.95	3.29	
	Postgraduate	7.43	5.35	
Marital Status	Married	5.39	4.30	0.939
	Single	5.39	2.82	
	Divorced	4.50	.71	
Nationality	Brazil	6.16	3.23	0.005
	Philippines	5.35	3.93	
	Other	5.58	2.81	
	China	2.14	2.12	
	Indian	3.57	3.41	
	Lebanese	5.71	4.35	

Attitude

Only a minority of participants (13.5%) accepted the possibility of contracting a Zika infection if traveling to or from the affected country while more than a third of the surveyed travelers (36.4%) denied this possibility and a similar portion (31%) reported being unaware of such issue. In regards to the issue of pregnancy and Zika infection, more than half (58%) stated that pregnant women must postpone their travel to any affected area while less than one-fifth (16%) disagreed with such a recommendation.

Practice

**Figure 3:**
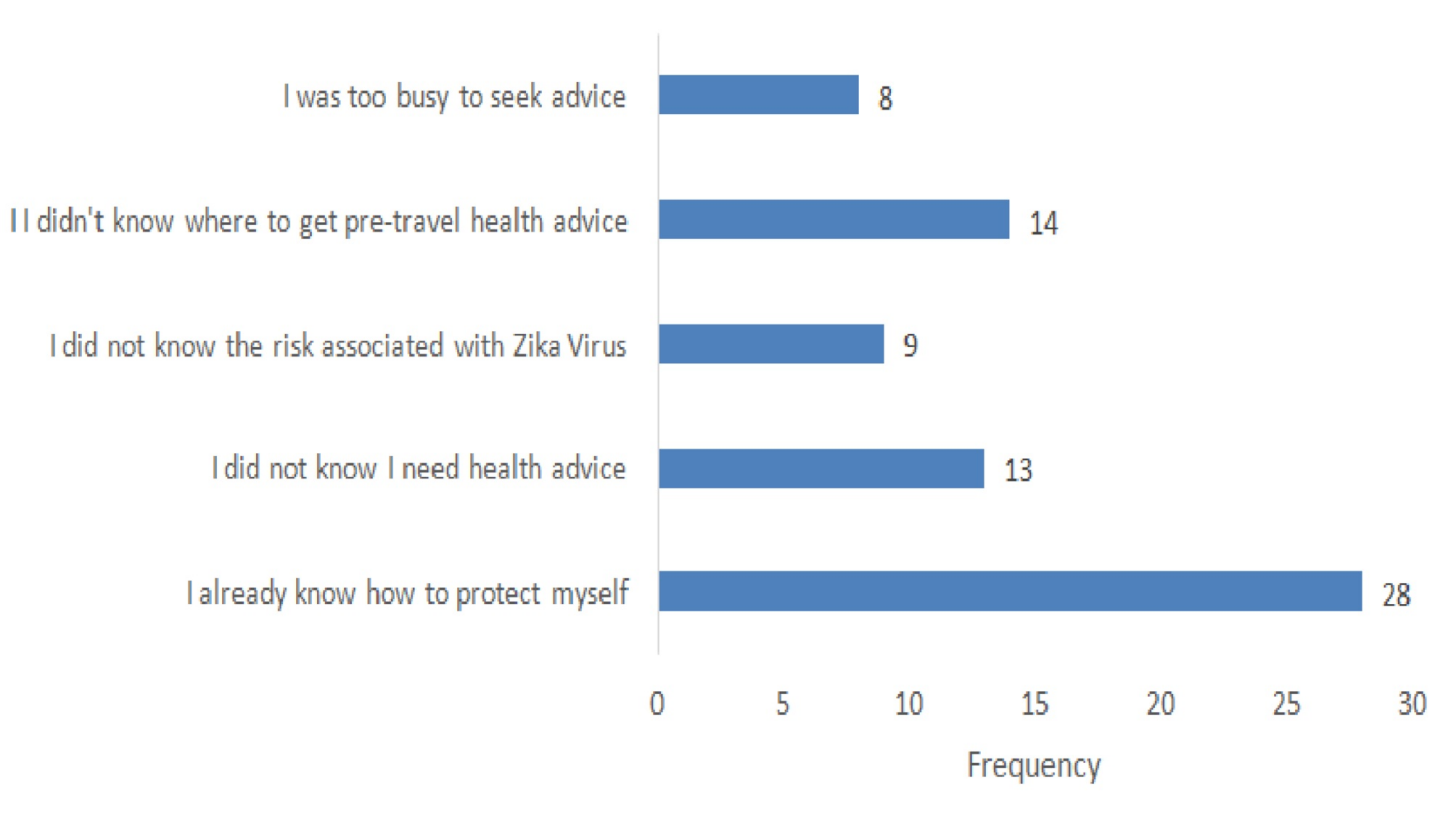
Reasons for travelers not seeking medical advice before travel

Almost a quarter of the travelers (24%) interviewed stated that they have sought pre-travel medical advice regarding their flight to Brazil. In addition, the internet constituted the main source of medical advice prior to travel (46%), followed by consulting a family physician (33.3%), a travel health specialist (20%), and family or friends (20%). On the other hand, among those who did not seek pre-travel medical advice (76%), more than a third (36%) reported that they already knew how to protect themselves while almost a fifth (18.4%) were unaware of where to get such advice. Moreover, a similar portion (17%) of the surveyed travelers did not know that they required pre-travel health advice, reflecting the lack of awareness among such travelers (Figure 3).

## Discussion

This study represents one of the first surveys to assess the knowledge, attitude, and practice of travelers regarding Zika disease in an airport setting, especially in the State of Qatar. A previous study was conducted at the university level in Qatar, with an online extension to reach the wider public in the country, and it revealed an inadequate level of knowledge regarding Zika [17]. Moreover, the results yielded from the current study are comparable to those from other surveys from different settings. For example, a similar survey conducted in the Selangor province of Malaysia revealed that almost three-quarters (71.5%) of the respondents possessed a good knowledge of the Zika viral disease, which reflects a high awareness level [18]. In this survey, it was found that more than two-thirds (69%) of the travelers correctly identified mosquito bites as a route of transmission for the Zika virus.

The current study has also reflected the influence of the media on the knowledge of travelers, as a majority of them (73%) had learned about Zika from such a source. Similarly, a survey on the knowledge and attitudes of Ecuadorian adults regarding Zika found that two-thirds of the respondents (68.2%) thought that the media impacted their decision to take an action to prevent the Zika virus, concluding the need for valid information sources to counter any misconceptions diffused through mass media [19].

This study also found that more than half of the travelers (58%) agreed with the health advice against the travel of pregnant women to Zika-affected areas. A similar survey in Greece on the knowledge and attitude of pregnant women towards Zika found that the majority (86%) of them reported it was unsafe to travel to Zika-affected countries during pregnancy [20]. In addition to that, the higher level of awareness among pregnant woman in the aforementioned study was attributed to their higher level of concern.

Regarding their practice and health-seeking behavior, only a quarter of the surveyed travelers (24%) sought pre-travel medical advice. Additionally, of those who pursued pre-travel health advice, only one-fifth consulted a travel health specialist. Thus, there exists a lack of awareness among travelers on the importance of seeking pre-travel health advice.

The current study has several limitations, one of them being the restricted time duration for conducting each survey because travelers had to board their planes, which may also have hindered potential respondents from participating in the study. Another limitation was that the majority of travelers interviewed were transit passengers and not originating from the State of Qatar and that the questionnaire provided was in the English language only. Thus, some potential respondents could not participate. An additional limitation to self-administered surveys is the self-reporting bias due to social desirability as well as acquiescence. However, the current paper represents the first study in the region to examine knowledge, attitude, and practice regarding Zika among travelers from and to affected areas. Moreover, the current study provided some baseline data; however, a larger and more comprehensive study will yield more representative and generalizable results. Also, this survey has provided an initial insight into the traveler’s knowledge, attitude, and practice regarding the Zika disease.

## Conclusions

Our study uncovered many potential areas for improvement and research, where larger studies are needed to further comprehend the travelers’ behavior from Qatar and other countries. Thus, interventional studies, particularly educational ones, are needed to increase the knowledge of travelers and the public on Zika. Moreover, follow-up studies (post-travel) of travelers, after equipping them with all the preventive measures and advice, is vital to evaluate the effectiveness of such measures. Finally, there is a need for the development of risk communication strategies and piloting them to detect their impact on the behavior of travelers, particularly for non-affected countries.
